# Prognostic scores for hepatocellular carcinoma: none is the winner

**DOI:** 10.1111/j.1478-3231.2009.01994.x

**Published:** 2009-04

**Authors:** Calogero Cammà, Giuseppe Cabibbo

**Affiliations:** 1 Cattedra di Gastroenterologia, Dipartimento Biomedico di Medicina Interna e Specialistica, University of PalermoPalermo, Italy; 2 Consiglio Nazionale delle Ricerche, IBIMPalermo, Italy

Cancer classification and indication of treatment are critical steps in the management of patients with hepatocellular carcinoma (HCC). The prediction of outcome is relevant to provide adequate information to patients and relatives, both at the time of treatment selection and after the application of therapy. Tumour staging describes the extent of an individual's tumour burden in the original primary organ and spread throughout the body, and other cofactors such as age or histological grade are only seldom considered. This is common to all malignancies and diseases. However, whereas for most neoplasms prognosis and treatment are largely dictated by tumour stage at the time of diagnosis, the scenario is more complex in patients with HCC.

It is well known that cirrhosis underlies HCC in most of the patients and the functional impairment of the underlying liver has a significant impact on prognosis, irrespective of the tumour stage. Moreover, liver function defines the capacity to indicate treatments with potential deleterious effects on the liver (1). This is well established in early tumours, in which resection may be contraindicated because of the deterioration of liver functional status and the same applies to patients with multifocal HCC that could be considered for locoregional therapies such as chemoembolization, in whom liver decompensation unequivocally argues against its indication.

Several staging systems have been developed for the classification of patients with HCC. The best known and assessed include eight systems based on clinical items: Okuda (2), Cancer of Liver Italian Program (CLIP) (3), Barcelona Clinic Liver Cancer (BCLC) (4), GRoupe d'Etude et de Traitement du Carcinoma Hépatocellulaire (GRETCH) (5), Tumour node metastasis (TNM) (6), Chinese University Prognostic Index (CUPI) (7), Japanese Integrated System (JIS) (8) and one molecular staging system, the estrogen receptor (ER) (9) classification proposed by Villa *et al.*

A major problem of the studies on prediction of patient outcomes from HCC on cirrhosis arises from a lack of prognostic tools able to adequately express the medical complexity of the syndrome. These tools could support an objective risk stratification in interventional studies, quality of care evaluation and allocation of health care resources. Unfortunately, no accurate mortality risk estimate is yet available to the hepatologist as decision-making support in order to avoid unethical and futile care of HCC patients. For instance, if the difference in overall survival with or without a new molecular-targeted therapy can be measured only in days or a few weeks, and liver transplantation is not an option, the need for intervention should be questioned.

The Okuda system (2) was developed about 20 years ago based on data from advanced HCC patients. It includes tumour size (occupying more or <50% of the liver) and three indicators of the severity of cirrhosis (ascites, serum albumin and bilirubin levels), but lacks important tumour factors, such as portal vein thrombosis, size and number of neoplastic lesions. A major issue of this system is that the assessment of tumour burden is applicable only to the advanced stage. The CLIP (3), GRETCH (5) and CUPI (7) systems were derived in different cohorts of patients with HCC identifying survival factors by multivariate analyses. The BCLC (4) staging system uses variables related to tumour stage, liver functional status and physical status. It is important to underline that the BCLC is not a prognostic model able to assess the mortality of HCC patients; instead, it is an allocation algorithm that, combining independent prognostic factors in four different stages (from A to D), links these stages with the best treatment option. The American Joint Committee on Cancer (AJCC) TNM staging system (6) considers the presence and the degree of severe cirrhosis or fibrosis to stratify outcome for every tumour (T) classification. The AJCC staging is the only one that is validated in patients treated with either hepatic resection or transplantation and is however rarely used in Europe. The JIS (8) is a new score system that includes two previous classifications: the TNM endorsed by the Union Internationale Contre le Cancer, mostly applied in Japan, and the Child–Pugh classification. It lacks external validation in western countries. Finally, a variant form of the wild-type estrogen receptor has been identified in some patients with HCC in which the receptor maintains constitutive transcriptional activity. Tumours containing this variant tend to be more aggressive, with shorter doubling times. The presence of variant estrogen receptors was a better predictor of an unfavourable prognosis compared with the CLIP and Barcelona. ER classification is not used routinely cause the need to perform nodule biopsy to test for the variant receptor (9).

Among all these prognostic scores, the BCLC and the CLIP are the most widely used and have been extensively validated in different geographical settings, although the adoption, in general, of one system appears to vary from country to country. We demonstrated in a large prospective unicentre study that the overall predictive ability of the BCLC and CLIP staging systems was not satisfactory and was not uniform for treated and untreated patients. None of the scoring systems provided a confident prediction of survival in individual patients (10). In our study, CLIP achieved the best discriminative capacity in the advanced untreatable cases whereas BCLC was the most able in predicting survival in treated patients. Similarly, a recent study (11) published in this journal confirmed that in a large cohort of about 4000 treated patients, the discriminatory ability of the CLIP score was modest for the early HCC stages. In particular, the CLIP-scoring system demonstrates significant interscore prognostic prediction. However, different prognostic weighting of four predictive factors may cause intragroup heterogeneity. The lower the CLIP score, the greater the intragroup difference. The authors' conclusion was that the CLIP-staging scoring system was a reasonable ordinal scale, but the clinician must be aware of the heterogeneity of mortality risk within a given score.

We fully agree with the conclusions of the Editorial by Ray Kim published in *Liver International*, who wrote that ‘No model is perfect and the utility of the current available prognostic models is that they are useful as a general guide’ (12). The homogeneity (precision) and discrimination of all the available prognostic scores are far from perfect and different lines of evidence suggest that none of these staging systems provided sufficient confidence for the prediction of outcomes in individual patients. Thus, no system has been recommended worldwide for clinical purposes (1) and a high degree of caution must be exerted in the clinical application of the existing models for outcome prediction in HCC patients.

Why, when validated internally or externally, does none of these staging systems provide sufficient confidence for the prediction of outcome in individual patients? Many factors, linked to the model's characteristics and the complexity of the disease, may contribute to a less than satisfactory performance. Possible biases are exemplified by heterogeneity in diagnostic criteria, time to diagnosis of HCC, time of referral or lead-time bias, true differences in case mix, presence and absence of the underlying cirrhosis and its aetiology and severity and systematic differences in the effectiveness of treatment algorithms. An important question concerning studies on prediction of mortality of HCC is that these studies were designed to combine a wide spectrum of patients – those with early, intermediate, advanced and end-stage HCC – pooling treatable and untreatable subjects. These studies compare the overall predictive ability of the different general patient models in the entire spectrum of patients to identify the best staging system rather than to allow the performance of those same models to be analysed in more homogeneous subgroups of patients with HCC (e.g. potentially treatable vs. untreatable subjects) (10).

When there is evidence that a given general model is not fully appropriate for outcome prediction in a particular clinical setting, a possible solution can be represented by the use of specialized models evaluating patient outcomes within specific allocation treatment groups (i.e. liver transplantation, percutaneous ablation, resection, transarterial embolization/chemoembolization and systemic therapies). However, a relevant shortcoming in predicting outcome in the setting of treated patients is due to the fact that the assessment of the benefit of the different treatments on overall survival by any staging system adds more complexity to the already high degree of epidemiological, biological and clinical heterogeneity of patients with HCC before treatment. Lastly, it is very difficult to evaluate the interaction between the different treatments and baseline prognostic variables and its own impact on overall survival. As a consequence, the ideal staging system able to link staging with treatment allocation and to predict the impact of treatment on life expectancy in the whole spectrum of patients with HCC today remains an elusive goal.

In the current issue of *Liver International*, Tandon *et al.* (13) performed a methodologically sound systematic review of the literature evaluating predictors of death in patients with cirrhosis and HCC, aiming to evaluate whether predictors today differ between patients with compensated and decompensated cirrhosis. A large number of studies (72), published as full-length papers, were quoted. This systematic review, with a total of 23 968 patients included, shows that there are very heterogeneous study designs. In fact, 71% of the studies were retrospective and only 29% were prospective. Only 57% of the studies enrolled patients consecutively. The best predictors of death in patients with cirrhosis and HCC are tumour and liver related. A total of 79 variables were evaluated in these studies. The most common independent predictors of death in HCC were portal vein thrombosis, tumour size, α-fetoprotein (AFP), the Child–Pugh class, bilirubin and the CLIP score.

When the 22 studies in whom 100% of the patients had cirrhosis were analysed, the most common predictors of death were the CLIP score, tumour size, the Child–Pugh class, tumour number, AFP and portal vein thrombosis. When patients were separated by advanced or nonadvanced tumour status, the most important predictors of death in patients with advanced tumours were portal vein thrombosis, AFP, bilirubin and lack of treatment.

Although the number of studies that included mostly compensated or decompensated cirrhotic patients was small, the first important conclusion of this review, which needs further confirmations, is that in the compensated patients, factors related to the tumour would be more important whereas in the decompensated patient, both liver- and tumour-related factors would be important.

The second and relevant finding of the study by Tandon *et al.* is to suggest that different predictors should be performed in specific patient populations to obtain a better prediction of death. We believe that this is an important attempt of customization of the available general prognostic models. A problem with this systematic review is that there are limitations in the data, which are beyond the authors' control. These include the lack of a standardized diagnostic criteria and staging procedures, the heterogeneity among studies in population characteristics and prevalence of compensated or decompensated cirrhosis. The authors are very fair in pointing out these limitations and the resultant limitations on the conclusions that may be drawn from summary data in the discussion.

Growing experimental evidences suggest that there are well-known factors not accounted for in the prognostic models that can have some impact on patient outcomes, both in early as well as in advanced stages.

Global gene expression profiling may be the most appropriate technology to explore its heterogeneous origin. In fact, application of gene expression profiling of HCC represents a promising progress in elucidating the molecular pathogenesis of HCC and in improving the prognostic prediction for HCC patients.

In conclusion, we truly need new and more accurate prognostic models that include important, although still unknown, biological variables and models that account for changes during the course of the disease.
